# Pressure- versus volume-limited sustained inflations at resuscitation of premature newborn lambs

**DOI:** 10.1186/1471-2431-14-43

**Published:** 2014-02-15

**Authors:** Graeme R Polglase, David G Tingay, Risha Bhatia, Clare A Berry, Robert J Kopotic, Clinton P Kopotic, Yong Song, Edgardo Szyld, Alan H Jobe, Jane J Pillow

**Affiliations:** 1 The Ritchie Centre, Monash Institute of Medical Research, Monash University, Clayton, Victoria 3168, Australia; 2 Neonatal Research, Murdoch Children’s Research Institute, Melbourne, Australia; 3 Neonatal Research, The Royal Women’s Hospital, Melbourne, Australia; 4 Neonatology, The Royal Children’s Hospital, Melbourne, Australia; 5 Department of Paediatrics, University of Melbourne, Melbourne, Australia; 6 Centre for Neonatal Research and Education, School of Paediatrics and Child Health, University of Western Australia, Perth, Australia; 7 School of Anatomy, Physiology and Human Biology, The University of Western Australia, Crawley, Western Australia 6009, Australia; 8 CAS Medical Systems Inc, Branford, CT, USA; 9 Department of Preventive Medicine, Icahn School of Medicine at Mount Sinai, New York, USA; 10 Cincinnati Children’s Hospital Medical Centre, Cincinnati, Ohio, USA; 11 Neonatal Clinical Care Unit, Women and Newborn Health Service, King Edward Memorial Hospital, Subiaco 6008, Western Australia, Australia

**Keywords:** Mechanical ventilation, Infant, newborn, Lung recruitment, Ventilation homogeneity, Variability

## Abstract

**Background:**

Sustained inflations (SI) are advocated for the rapid establishment of FRC after birth in preterm and term infants requiring resuscitation. However, the most appropriate way to deliver a SI is poorly understood. We investigated whether a volume-limited SI improved the establishment of FRC and ventilation homogeneity and reduced lung inflammation/injury compared to a pressure-limited SI.

**Methods:**

131 d gestation lambs were resuscitated with either: i) pressure-limited SI (PressSI: 0-40 cmH_2_O over 5 s, maintained until 20 s); or ii) volume-limited SI (VolSI: 0-15 mL/kg over 5 s, maintained until 20 s). Following the SI, all lambs were ventilated using volume-controlled ventilation (7 mL/kg tidal volume) for 15 min. Lung mechanics, regional ventilation distribution (electrical impedance tomography), cerebral tissue oxygenation index (near infrared spectroscopy), arterial pressures and blood gas values were recorded regularly. Pressure-volume curves were performed *in-situ* post-mortem and early markers of lung injury were assessed.

**Results:**

Compared to a pressure-limited SI, a volume-limited SI had increased pressure variability but reduced volume variability. Each SI strategy achieved similar end-inflation lung volumes and regional ventilation homogeneity. Volume-limited SI increased heart-rate and arterial pressure faster than pressure-limited SI lambs, but no differences were observed after 30 s. Volume-limited SI had increased arterial-alveolar oxygen difference due to higher FiO_2_ at 15 min (p = 0.01 and p = 0.02 respectively). No other inter-group differences in arterial or cerebral oxygenation, blood pressures or early markers of lung injury were evident.

**Conclusion:**

With the exception of inferior oxygenation, a sustained inflation targeting delivery to preterm lambs of 15 mL/kg volume by 5 s did not influence physiological variables or early markers of lung inflammation and injury at 15 min compared to a standard pressure-limited sustained inflation.

## Background

Initial resuscitation of preterm infants aims to establish a functional residual capacity (FRC) and facilitate initiation of gas-exchange within the immature lung. However, the initiation of ventilation after preterm birth may be a critical period of susceptibility for the development of lung and brain injury [1-4].

Sustained inflation at birth is practiced in some centers for early establishment of FRC [5,6]. A sustained inflation is recommended for the initial ventilation of apneic term and preterm infants in the recent European Resuscitation Council Guidelines [7]. An initial inflation sustained for 20 s fully aerates the preterm rabbit lung prior to the onset of tidal ventilation [8]. A sustained inflation also facilitates establishment of pulmonary blood flow immediately after birth and improves cerebral blood flow stability in preterm lambs compared to preterm lambs resuscitated without a sustained inflation [9]. The optimal way to deliver a sustained inflation is unknown.

Current neonatal resuscitation guidelines published by the European Resuscitation Council and American Heart Association, suggest that the initial inflations should be given by constant application of a predetermined inflation pressure [7,10]. However, the lung volume achieved with a set pressure is dependent upon the mechanics of the respiratory system, the maturational stage of lung development and the volume of lung liquid remaining within the air spaces. Thus, application of a constant pressure may have variable efficacy in establishing a functional residual capacity. Acute over-distension resulting from a high sustained inflation volume delivered with a constant pressure could have injurious effects on the preterm lung and brain, whereas a low inflation volume would be ineffective in aerating the fluid-filled lung.

An alternative approach to sustained inflations would be to target delivery of a defined volume for the initial inflation. Whereas a defined initial delivered volume would potentially achieve more consistent volume inflation of the non-aerated lung, the inflation pressure required to achieve the predetermined volume would be variable and could result in exposure of the immature lung to potentially injurious high static inflating pressures, and possibly pneumothoraces.

We aimed to understand whether delivery of sustained inflations should be pressure- or volume-limited. Specifically, we asked how the method of sustained inflation influenced the homogeneity of aeration, the consistency of the functional residual capacity achieved immediately at the end of the sustained inflation, and the up-regulation of early markers of lung injury. We hypothesized that a sustained inflation that targets a preset delivered volume/kg birth weight will provide a more consistent FRC and more homogeneous aeration than is achieved using a pressure-limited sustained inflation, potentially reducing lung injury.

## Methods

All experimental procedures were approved by the animal ethics committee of The University of Western Australia, in accordance with the National Health and Medical Research Council (Australia) Australian code of practice for the care and use of animals for scientific purposes (7^th^ Edition, 2004).

### Surgical preparation

Surgery was performed on anesthetized pregnant ewes, bearing single or twin fetuses, at mean (SD) 131 ± 0.8 d gestation (term is ~147 d). The fetal head and neck were exposed via hysterotomy for surgical insertion of occlusive polyvinyl catheters into a carotid artery and jugular vein. Carotid arterial and jugular venous pressures were recorded digitally (1 kHz: Powerlab, ADInstruments: Castle Hill, Australia). The fetal trachea was intubated orally (4.5 cuffed tracheal tube, Portex Ltd, UK). Standardized intratracheal suction (same depth and duration) was performed to control the level of lung liquid remaining after birth. Electrical Impedance Tomography (EIT; Goe-MF II EIT system, Carefusion, Hoechberg, Germany) electrodes were evenly spaced circumferentially around the chest at the level of the axillae for measurement of regional lung aeration as described previously [11-14].

Immediately after instrumentation, lambs were delivered surgically, dried, weighed and ventilated according to their assigned protocol (see below). Propofol (0.1 mg/kg/min, Repose™, Norbrook Laboratories, Victoria Australia) and remifentanil (0.05 μg/kg/min, Ultiva™, Glaxo Smith Kline, Victoria, Australia) were administered by continuous infusion (umbilical venous catheter) for anesthesia, analgesia and suppression of spontaneous breathing. Gas exchange and acid base balance was monitored by blood gas analysis at 5 min intervals (Rapidlab 1265, Siemens Healthcare Diagnostics, Vic, Australia).

### Sustained inflation and ventilation strategies

The lambs were ventilated in the prone position. Lambs were randomized to receive a total of 20 s of sustained inflation delivered either as:

1) a pressure-limited sustained inflation (PressSI) with a continuous ramped increase in inflating pressure to a maximum of 40 cmH_2_O by 5 s, which was maintained for a further 15 s; or

2) a volume-limited sustained inflation (VolSI) with inflating pressure adjusted to deliver a inflation volume of 15 mL/kg by 5 s, which was maintained for a further 15 s.

Sustained inflations were delivered with a fractional inspired oxygen content (FiO_2_) of 0.3. After the sustained inflation, all lambs received a programmed *V*_T_ of 7 mL/kg, with a positive end-expiratory pressure (PEEP) of 5 cmH_2_O (FlexiVent, Scireq, Montreal, Canada) for a total ventilation period of 15 minutes. Ventilation was with warmed, humidified gas with an initial FiO_2_ of 0.3, which was adjusted to targeted pre-ductal transcutaneous oxyhemoglobin saturation (SpO_2_, Nellcor OxiMax N65, Tyco Healthcare, Australia) of 90-95% from 5 minutes of age.

### Measurements and calculations

Near infrared spectroscopy (Fore-Sight Tissue Oximeter, CAS Medical Systems Inc., Branford, CT USA) was used for continuous recording of cerebral oxygenation using the small sensor, which was placed over the fronto-parietal region and covered with a light-proof dressing. Cerebral oxygenation was expressed as a tissue oxygenation index (SctO_2_, %) at 0.5 Hz.

Arterial oxygenation was assessed by calculating the alveolar-arterial difference in oxygen (AaDO_2_). Cerebral oxygen extraction was calculated as C(a-v)O_2_/CaO_2_, where [C(a-v)O_2_] is the difference in carotid arterial and jugular venous oxygen content. Arterial or venous oxygen content (CaO_2_ and CvO_2_ respectively) was determined as (1.39 · Hb · SaO_2_ /100) + (0.003 · PaO_2_) (33), where Hb is the hemoglobin concentration (g/dL), and SaO_2_ is the arterial oxyhemoglobin saturation.

Partitioned measurements of respiratory mechanics were obtained using the low-frequency oscillation technique at 5 minute intervals, immediately following blood gas measurements: pressure (*P)* and volume (*V*) measured during an optimized ventilator waveform (average tracheal tidal volume 7 mL/kg, 0.5 – 13 Hz) [15] delivered by the FlexiVent were used to calculate input lung impedance. The constant phase tissue model [16] was fitted to the impedance spectra to determine a frequency independent airway resistance (*R*_aw_), constant-phase tissue damping (*G*, similar to tissue resistance) and tissue elastance (*H)*.

Relative impedance (Z) was measured by EIT at 25 Hz and analyzed offline (AUSPEX V1.6, Carefusion). To isolate the end-expiratory volume (EEV), the trough of each respiratory cycle was determined after low-pass filtering the impedance signal to the respiratory domain [11-13,17]. The EIT data were divided into three regions of interest (ROI); the global and gravity-dependent (ventral) and non-dependent (dorsal) hemithoraces. Relative change in EEV within each ROI was then expressed as a percentage of the vital capacity for that ROI (Z %_VCroi_). Vital capacity was defined as the difference in impedance at 0 and 40 cmH_2_O in a ROI during a post mortem super-syringe static pressure-volume curve [12,18,19].

### Postmortem analyses

At 15 minutes the lambs were heavily anesthetised prior to ventilation with 100% O_2_ for 2 minutes, after which the tracheal tube was clamped for 3 minutes to facilitate lung collapse by oxygen reabsorption. This process allows for the lungs to become atelectatic prior to static measurement of lung compliance [20]. Lambs were euthanized with intravenous sodium pentobarbitone (100 mg/kg) and an *in situ* post-mortem super-syringe static pressure-volume curve was generated [21].

Bronchoalveolar lavage (BAL) was obtained by triplicate washouts of the left lung [22]. Total protein content of the BAL was determined by the Lowry method [23].

Lung pieces were cut from the right lower lobe and immediately frozen in liquid nitrogen for later quantitative real-time polymerase chain reaction (qRT-PCR) analysis of early markers of lung injury including Connective Tissue Growth Factor (CTGF), Cysteine-rich 61 (CYR61) and Early Growth Response protein 1 (EGR1) mRNA, as described previously [24]. qRT-PCR results were analyzed using the 2^-ΔΔCT^ method [25].

### Statistical analysis

Fetal blood gas variables and mRNA cytokine expression data were compared between groups using a Students t-test (SigmaPlot v12.0, Systat Software Inc). Postnatal assessments were compared using two-way repeated measures ANOVA using time and group assignment as the two factors, and subject number as the repeated measure. Holm-Sidak multiple comparisons posthoc test was used to determine differences between groups. Statistical significance was accepted as p < 0.05. Data are presented as mean (SEM) for parametric data or median (interquartile range) for non-parametric data.

## Results

### Fetal characteristics

There were no differences in delivery order or fetal weight, sex, umbilical cord arterial blood gas status at delivery, with the exception of arterial pH, which was lower in PressSI lambs (Table 1).

**Table 1:** Birth characteristics and fetal umbilical arterial blood-gas variables at delivery

	**PressSI**	**VolSI**
Number	8	7
Male n (%)	3 (38)	4 (57)
Birth order 1st n (%)	4 (50)	6 (86)
Birth weight (kg)	3.04 (0.13)	3.02 (0.14)
pHa	7.11 (0.02)	7.24 (0.02)*
*P*aCO_2_	71.5 (3.8)	59.9 (5.1)
*P*aO_2_	16.1 (1.8)	20.9 (1.9)
Hct (%)	35.9 (4.7)	33.8 (2.7)

Arterial pH (pHa), hematocrit (Hct) and partial pressure of arterial (*P*a) carbon dioxide (CO_2_) and oxygen (O_2_). * indicates significant difference (p < 0.001).

### Arterial blood-gas and ventilation variables

**Figure 1:**
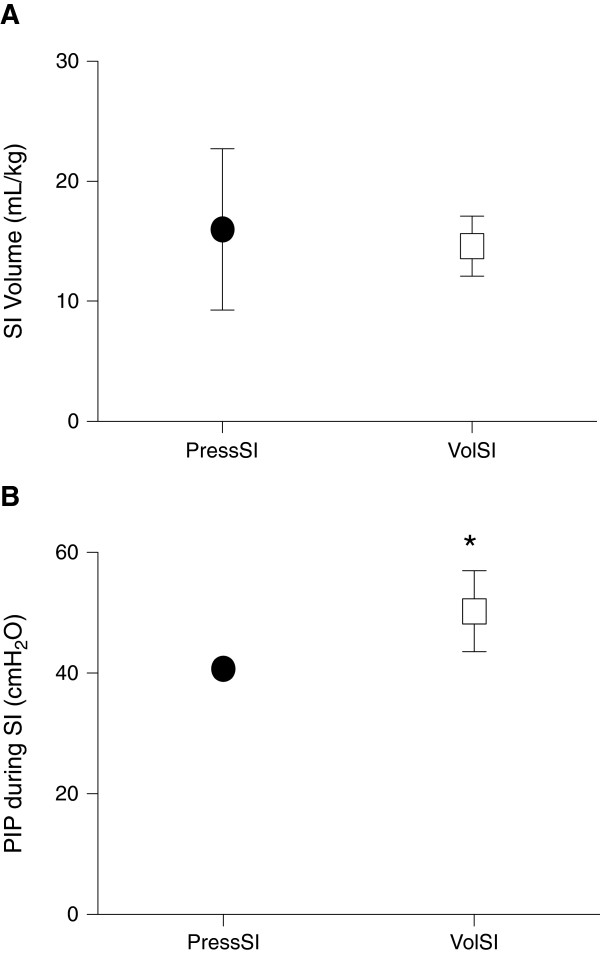
**Volume and pressure measurements at completion of the 20 s sustained inflation (SI). A)** Delivered volume (SI Volume at 20 s) and **B)** peak inspiratory pressure (PIP) during pressure-limited (PressSI; black circles) and volume-limited (VolSI; open squares) sustained inflations. * indicates significant difference (p<0.05).

Mean volume (per kg bodyweight) recruited at the end of the 20-s sustained inflation was not different between groups although higher variability was evident in volumes delivered to PressSI compared to VolSI lambs: mean (SD) of inflation volume was 16.0 (6.7) mL/kg for PressSI lambs versus 14.6 (2.5) mL/kg for VolSI lambs (Figure 1A). Peak pressure during sustained inflation was significantly higher in the VolSI lambs (50.2 (6.7) cmH_2_O) versus PressSI lambs (40.8 (1.2) cmH_2_O; p = 0.004; Figure 1B), but was not different between groups over the subsequent 15 min ventilation. Median (25^th^, 75^th^ centile) pressure over the duration of the sustained inflation was lower in the VolSI group (36.6 (32.2, 42.7) cmH_2_O) compared to the PressSI group (40.0 (39.3, 40.0) cmH_2_O) (p = 0.002).

**Figure 2:**
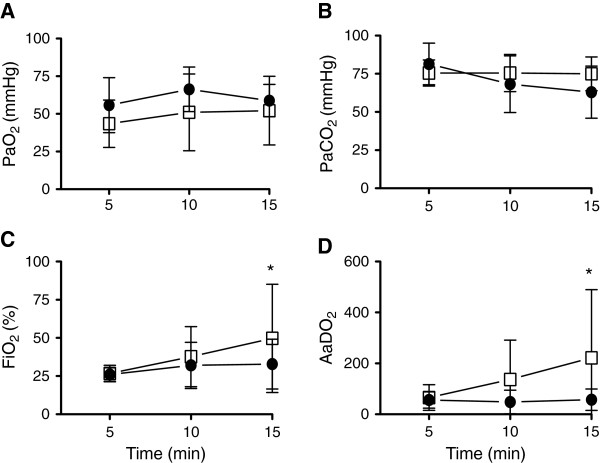
**Temporal measures of gas exchange over 15 minutes following pressure or volume limited SI: A)** Partial pressure of arterial (Pa) oxygen (PaO2), **B)** carbon dioxide (PaCO2), **C)** percentage of inspired oxygen (FiO2) and **D)** alveolar-arterial difference in oxygen (AaDO2) in pressure-limited (PressSI; black circles) and volume-limited (VolSI; open squares) sustained inflations. * p<0.05.

*P*aO_2_, *P*aCO_2_ and SaO_2_ were not different between groups throughout the 15 min ventilation period (Figure 2A&B). FiO_2_ and consequently AaDO_2_ were increased in VolSI lambs compared to PressSI (p = 0.01 and p = 0.02 respectively) at 15 minutes (Figure 2C & D). Regional cerebral oxygenation and cerebral oxygen extraction were not different between groups immediately after the sustained inflation, or at any time during ventilation between groups (data not shown: p = 0.850 and 0.126 respectively).

### Hemodynamic measurements

**Figure 3:**
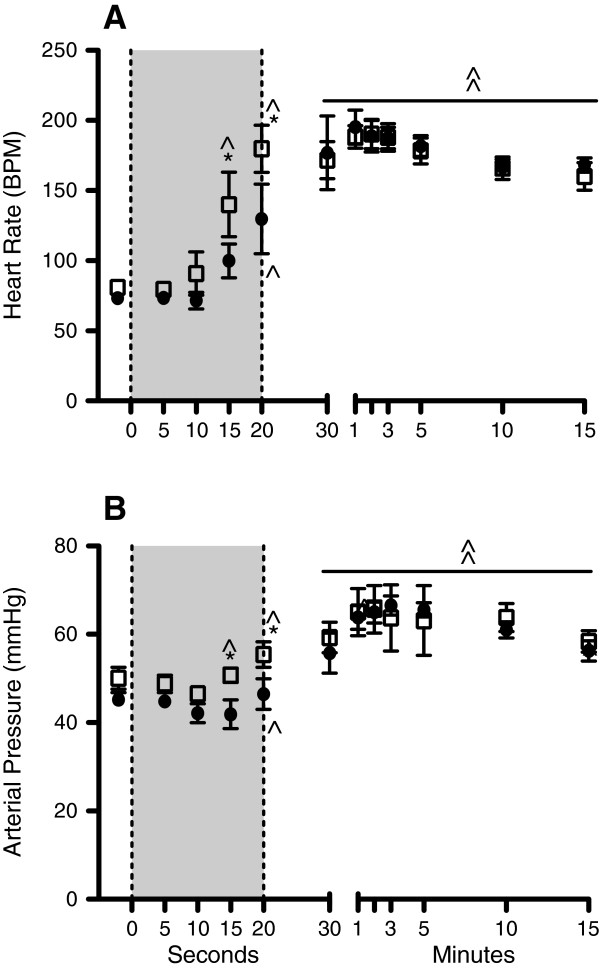
**Temporal changes in cardiovascular responses to Pressure and Volume limited SI: A)** Heart rate and **B)** arterial blood pressure during the sustained inflation (shaded region) and upon subsequent ventilation in pressure-limited (PressSI; black circles) and volume-limited (VolSI; open squares) sustained inflations. * indicates significant difference between Press SI and Vol SI groups (p<0.05). ^ indicates significant difference from fetal (F) value within each group. BPM – beat/min.

Heart rate and arterial pressure during the VolSI were significantly increased above the fetal value by 15 s and remained higher for the remainder of the study (Figure 3). Heart rate and arterial pressure during the PressSI were significantly increased above the fetal value by 20 s and remained higher for the remainder of the study (Figure 3). Heart rate and arterial pressure in VolSI lambs was significantly higher at 15 s and 20 s compared to PressSI lambs (Figure 3), but not thereafter (Figure 3). Central venous pressure (p = 0.314) was not different at any stage of the SI or subsequent ventilation strategy.

### Regional aeration

**Figure 4:**
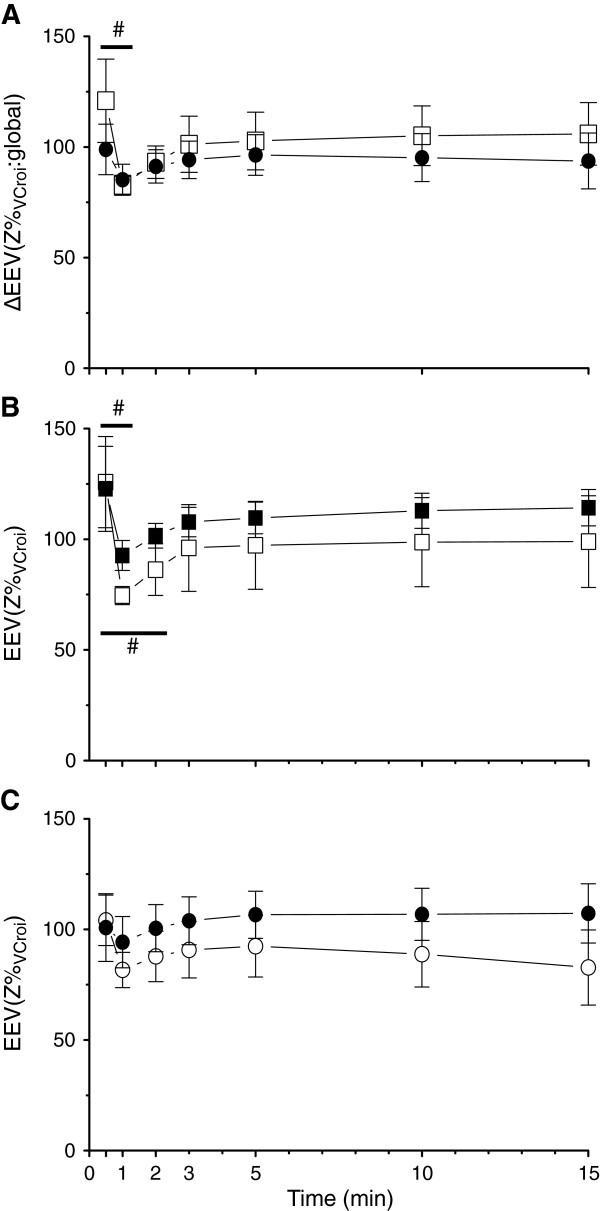
**Effect of pressure- and volume-limited SI on regional lung impedance determined by electrical impedance tomography: A)** Global change in end-expiratory lung volume (∆EEV) expressed as percentage change from the total lung volume determined at autopsy, in pressure-limited (PressSI; black circles) and volume-limited (VolSI; open squares) sustained inflations. Regional end-expiratory lung volume in dependent (closed shapes) and non-dependent (open shapes) in PressSI **(B)**, and VolSI **(C)**. # indicates significant difference between 20 s and 1 minute.

The change in global EEV, expressed as a percentage of vital capacity, was not different between groups at the completion of the sustained inflation or after subsequent ventilation (p = 0.546; Figure 4A). At the completion of the sustained inflation, the change in global EEV was higher in both groups than at any other time of the ventilation, including when the lungs were inflated to 40 cmH_2_O during a static pressure-volume measurement immediately after lambs were killed at study completion. Global end-expiratory lung volume significantly decreased after the sustained inflation in the VolSI group, from a mean (SD) 121.0 (49.7) Z %_VC,roi_ at 30 s to 82.8 (11.8) Z %_VC,roi_ at 60 s (p = 0.007), but not in the PressSI group (p = 0.984).

There were no significant differences in regional EEV within the dependent or non-dependent thorax between groups (Figure 4B & C). There was a significant reduction of EEV between the end of the SI and 1 and 2 minute time points within the dependent lung (p < 0.001) and between the end of the SI and 1 minute time point in the non-dependent lung (p = 0.045) within the VolSI group (Figure 4B). No significant loss of EEV was observed in the PressSI group in either lung region.

### Partitioned forced oscillatory mechanics

**Figure 5:**
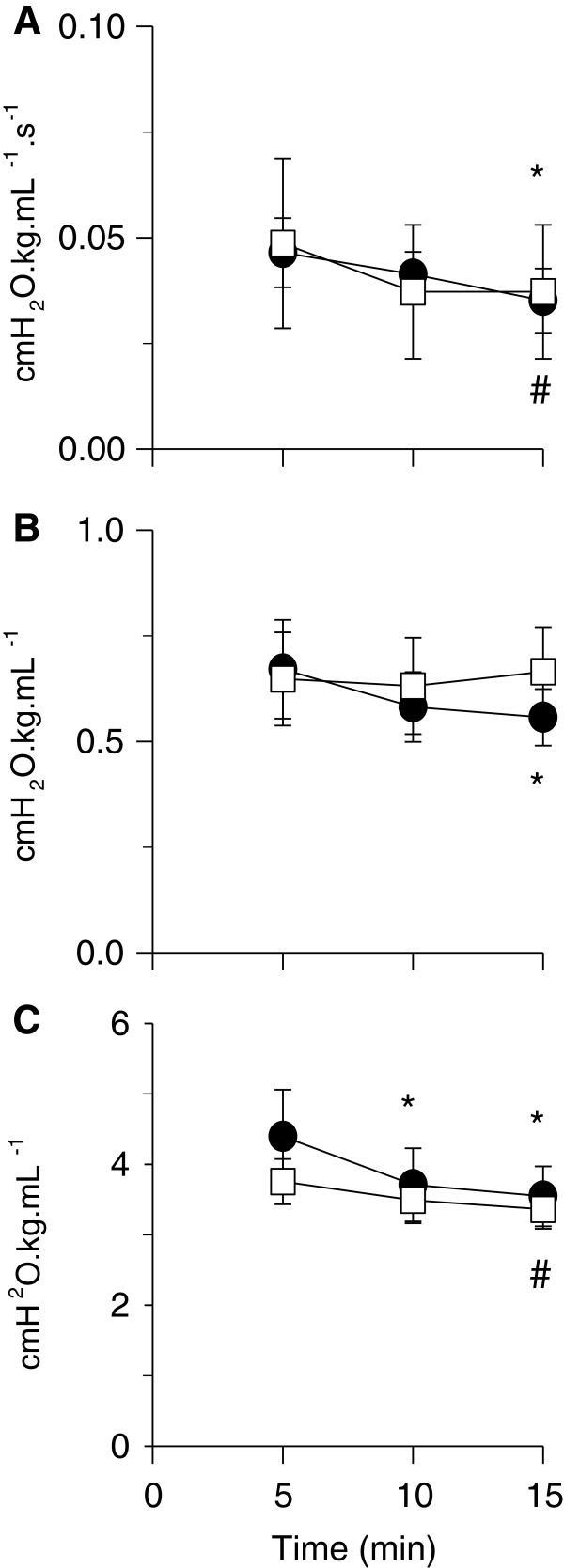
**Temporal changes in lung mechanics associated with pressure- and volume-limited SI: (A)** Newtonian (airway) resistance (Raw); **(B)** tissue damping (G); and **(C)** tissue elastance (H) in pressure-limited (PressSI; black circles) and volume-limited (VolSI; open squares) sustained inflations. * significantly different to 5 min for PressSI; # significantly different to 5 min for Vol SI. There were no intergroup differences in mechanics.

Newtonian *R*_aw_ decreased with time (Figure 5A p = 0.002) but there were no differences between PressSI and VolSI groups (Figure 5A). Tissue damping (G, ≈ tissue resistance) was lower at 15 min than at 5 min in the PressSI group, whilst no change was observed in the VolSI group (Figure 5B). Tissue elastance (*H*) decreased (ie compliance increased) with increasing time from delivery (p < 0.001). Compared to the 5 min measurement, tissue elastance was significantly lower at 10 min and 15 min in the PressSI group, and at 15 min in the VolSI group (Figure 5C). There was no main effect of group for any of the oscillatory mechanics outcomes.

### Post-mortem assessments

Static lung compliance, as determined by post-mortem pressure volume curves, was not different between groups (p = 0.598: Data not shown). Lung injury markers of total protein in the bronchoalveolar lavage fluid and mRNA expression of EGR, CYR61 and CTGF were not different between groups (Table 2).

**Table 2:** Markers of lung injury determined by qRT-PCR

	**PressSI**	**VolSI**	**p**
Total protein in BALF (ug/mL)	1740 ± 346	1664 ± 485	0.45
EGR1	1.0 ± 0.7	0.8 ± 0.9	0.33
CYR61	1.0 ± 2.0	0.6 ± 1.9	0.34
CTGF	1.0 ± 1.0	1.1 ± 1.1	0.69

BALF – Bronchoalveolar lavage fluid; EGR1 – early growth response protein 1; CYR61 – cysteine-rich 61; CTGF – connective tissue growth factor relative mRNA expression. mRNA expression is shown as median ± range expressed relative to the PressSI group.

## Discussion

Sustained inflations may promote rapid establishment of FRC during the initial resuscitation and ventilation of preterm and term infants [8,26]. However, the most effective and least injurious way to deliver a sustained inflation is not well understood. Three randomized controlled trials investigated the use of pressure limited SIs in preterm infants [27-29]: only the study by te Pas and colleagues showed improved clinical outcome, including decreased need for intubation in the first 72 h, shorter duration of ventilatory support and reduced bronchopulmonary dysplasia. No studies investigating the use of volume limited sustained inflations in the delivery room are reported, likely due primarily to the limitations of current delivery room resuscitation devices. We investigated whether a pressure- or volume-limited sustained inflation was more beneficial for the establishment of FRC, improving aeration homogeneity and reducing lung inflammation and injury. We observed worse arterial oxygenation at 15 min in volume-limited sustained inflations, but no differences in regional or total end expiratory lung volumes, lung injury, or cerebral oxygenation variables between the two sustained inflation strategies.

The volume delivered to a preterm lung exposed to a predetermined pressure is determined by the lung compliance, as well as the duration of the inflation relative to the time-constant (resistance x compliance) of the respiratory system. The large volume of fetal lung fluid present in the preterm ovine airways at birth results in significantly elevated respiratory resistance compared to the air-filled lung, and consequently a prolonged time-constant of the respiratory system at delivery. Giving the same duration and depth of suctioning of the intubated lamb airway equalized the residual lung fluid volume between all lambs. Thus, it is likely that the predominant factor determining the variability of the delivered tidal volume at the end of the sustained inflation was lung compliance. While the mean tidal volume delivered by the sustained inflation was similar between strategies, considerably more variation in the sustained inflation volume was observed in PressSI lambs compared to the VolSI groups. Although VolSI lambs should have had no variability in sustained inflation volume, some of the lambs within the VolSI group did not reach the targeted 15 mL/kg as the peak PIP was limited to 50 cmH_2_O to minimize pneumothoraces. The target of 15 mL/kg was chosen as this was demonstrated previously to be the functional residual capacity of lambs of this gestation [9] and was also based on our best approximation of the likely average volume delivered during the PressSI group in preliminary studies.

Our finding of increased variability in delivered volume using a pressure-limited SI at initiation of ventilation is consistent with recent reports of variable tidal volumes (0-30 mL) achieved with pressure-limited resuscitation of preterm infants [30-32]. Whereas these measured tidal volumes in preterm infants were confounded by the influence of facemask leak, our measurements were free of leak due to the use of tracheal tubes with inflated cuffs. Given the known association between serial high tidal volumes at initiation of ventilation and development of lung injury in the newborn lung [3,33-36], we hypothesized that the use of a set sustained inflation pressure may inadvertently initiate lung injury as a result of high delivered volumes in preterm subjects with compliant lungs.

**Figure 6:**
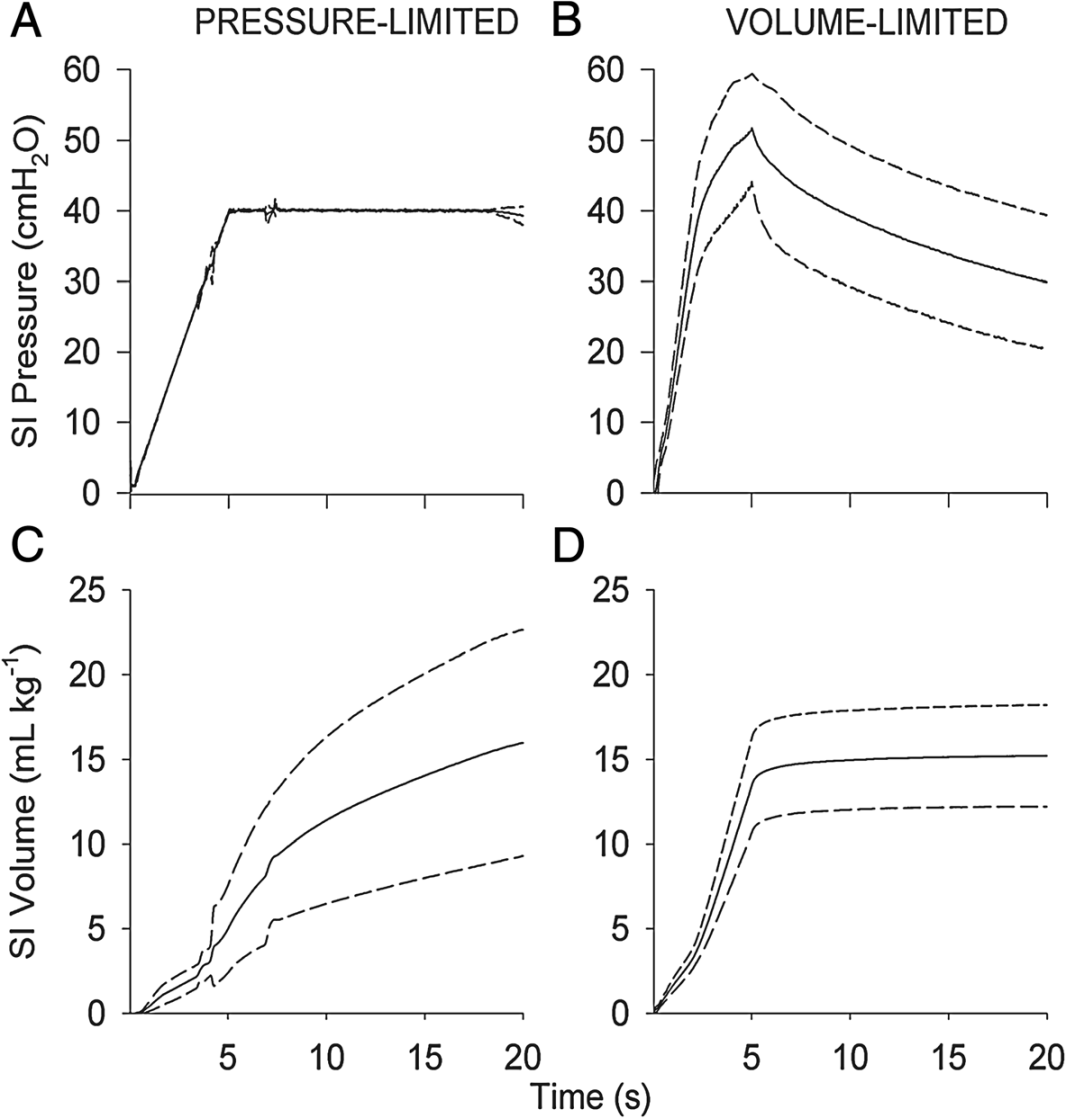
**Instantaneous changes in pressure and volume during the sustained inflation:** Pressure **(panels A and B)** and volume **(panels C and D)** during pressure limited (left panel) and volume-limited (right panel) sustained inflations. Solid line represents mean, dashed line indicates SD of mean, varying with time.

In contrast, a pressure-limited sustained inflation may be ineffective in achieving a functional increase in lung volume in the presence of poorly compliant lungs. We showed that a volume-limited sustained inflation overcomes the impact of compliance, to achieve a target inflation volume more reliably. However, the more consistent volume delivered with a volume-limited sustained inflation was achieved at the cost of more variable and on average 10 cmH_2_O higher peak inspiratory pressures than were required during pressure-limited sustained inflation. Thus, a critical question is whether excessive inflation due to volume stretch, or excessive pressure to achieve appropriate inflation, is the most injurious strategy. Barotrauma is also known to cause injury in preterm lungs leading to BPD [37], however it is unlikely that barotrauma was responsible for the difference in oxygenation and early fall in FRC in the VolSI group as evidence by the pattern of pressure and volume delivery for each group (Figure 6): despite higher peak pressures in the VolSI group, peak pressures were achieved only momentarily with a fall in sustained inflation pressure after the first five seconds with lower median pressure over the course of the sustained inflation in VolSI group. The fall in inflation pressure may have contributed to the impaired oxygenation and early (<2 min) fall in the end-expiratory volume noted in the VolSI group on analysis of EIT recordings. Whereas no further volume recruitment occurred after the first 5 s in the VolSI group, recruitment was achieved more slowing and was ongoing in the PressSI group at the end of the 20 s sustained inflation period. for the remaining 15 s), the pattern of pressure and volume delivery to the lung differed between the two groups (Figure 6). Volume recruitment was achieved slowly with the PressSI group whereas the rapid volume delivery in the VolSI group resulted in very brief exposure to high inflation pressure with a subsequent fall off in inflation pressure. This pattern of rapid inflation (as opposed to rapid then sustained pressurization), may explain the subsequent fall in end-expiratory volume (EEV) noted on EIT analysis. However, this fall in EEV within the VolSI group may simply be due to a reduction in pressure of ~10 cmH_2_O when the modality was switched to volume guarantee targeting 7 ml/kg. An alternative approach to volume-limited SI that aimed to achieve target volume at 20 s rather than at 5 s may have resulted in lower peak pressures but more sustained pressure throughout the recruitment process. Arguably, a target inflation volume delivered more slowly would achieve more homogeneous distribution with a lower risk of overdistension of fast time-constant lung units.

Both SI strategies significantly elevated HR and arterial pressure above the fetal values by the end of the sustained inflation (20 s) suggesting that both SI strategies were suitable at transitioning the fetal circulation to that of the newborn as shown previously [9,38]. Interestingly, the VolSI group elevated HR and arterial pressure faster (by 5 s) than the Press SI group (Figure 3). This likely occurred due to the higher lung volume of the lung during the first 10 s in VolSI lambs, which would clear lung liquid faster, increase aerated regions of the lung, establish functional residual capacity and trigger the hemodynamic transition at birth [8,26,38]. It is unlikely that the more rapid hemodynamic transition observed in the VolSI group would infer long-term benefits.

The 131 d lamb is approximately similar to 34-36 w GA in the human infant – a group that is still prone to respiratory distress due to surfactant deficiency. We demonstrated previously that naïve (non-steroid) lambs at 130-133 d gestation are surfactant deficient [39] with a negligible pool of saturated phosphatidyl choline and with little chance of postnatal survival without antenatal exposure to corticosteroids and significant postnatal intervention. The initiation of resuscitation of preterm lambs with even a few serial high volume breaths is sufficient to initiate a pulmonary and systemic inflammatory response, potentially leading to adverse long-term consequences of neonatal lung disease [3,33-36]. Given the short study period, we investigated only early markers of lung inflammation and injury to determine whether volume- or pressure-limited sustained inflations initiated lung inflammation and injury. Total protein content in the bronchoalveolar lavage fluid is a measure of lung injury [40]. We observed no differences in total protein content between SI strategies. EGR-1, CTGF and CYR-61 mRNA expression are sensitive early markers of the degree of lung injury, increasing within 15 min after lung injury induced by ventilation of preterm lambs, with amplified expression in response to injurious ventilation [24]. Our failure to observe differences in mRNA levels of any these early-response genes suggests that there was no difference between pressure- or volume-limited sustained inflations in early markers of lung inflammation or injury. It is possible that the 15 min exposure was too short to affect a demonstrable rise in mRNA expression of any early response markers. However, longer duration studies are also compromised for assessment of lung injury, as it is difficult to delineate the inflammatory response to the sustained inflation from the inflammatory response to the subsequent ventilation strategy. We did not collect lung for histological assessment of injury, but given the short duration of ventilation, it is unlikely that structural differences would be evident by 15 min.

### Limitations of the study

This study has some limitations not already discussed. Lambs were anaesthetized, hence spontaneous breathing was inhibited. Lack of spontaneous breathing may have influenced the outcome of the sustained inflation strategies. However, it is unlikely that infants would breathe during a sustained inflation due to the activation of the Hering-Breuer reflex from pulmonary stretch receptors in the smooth muscle of the lung, which prevents over-stretching of the lung by inhibiting inspiration [41]. The volume of fetal lung fluid recovered was also not measured. Differences in residual fetal lung liquid may alter the response to a sustained inflation.

EIT and NIRS measure only a small section of the organ of interest; neither measurement is necessarily representative of changes within the whole organ being examined. Therefore, the EIT and NIRS findings need to be extrapolated to the whole organ with caution. The limitations of EIT to measure relative changes in thoracic volume have been well described previously [42]: in particular, the different chest shapes of the preterm sheep lung and human may influence image reconstruction [42]. Alternatives to EIT, such as computerized tomography [19] and phase contrast X-ray imaging [43] are available but neither were clinically or technically practical for this study. The finding that global EEV was higher in all groups at the end of the sustained inflation relative to the total lung capacity determined during a post mortem super-syringe static pressure-volume curve is especially curious. One explanation for this discrepancy is a temporal change in the impedance signal over the study period, highlighting a potential limitation of calibrated EIT to track changes in distending lung volume.

## Conclusion

We demonstrated the efficacy of volume-limited sustained inflations for lung recruitment in preterm lambs. Sustained inflation procedures resulted in significant variability in either the delivered volume (PressSI) or pressures (VolSI). The VolSI strategy increased heart rate and arterial pressure sooner than the PressSI strategy, but had worse oxygenation at 15 min. There were no differences observed in physiological variables after 30 s or early markers of lung inflammation and injury between different sustained inflation strategies. While our findings suggest that both volume- and pressure-limited sustained inflations will obtain similar outcomes in preterm populations, the results need to be verified in preterm infants once devices suitable for delivery of volume-limited sustained inflations are available for infants.

## Abbreviations

BAL: Bronchoalveolar lavage; EIT: Electrical impedance tomography; FRC: Functional residual capacity; PEEP: Positive end expiratory pressure; PIP: Peak inspiratory pressure; SI: Sustained inflation; VT: Tidal volume.

## Competing interests

RK and CK were employees of Casmed Medical Systems. The remaining authors have no financial or non-financial competing interests to declare.

## Authors’ contributions

JP conceived the study. JP, GP, DT, RB, RK, CK, ES and AH contributed to the design, coordination and implementation of the animal studies, data acquisition and analysis and critical revision of the manuscript. YS and CB assisted with surgery, performed the molecular and protein assessments, data analysis and revision of the manuscript. All authors read and approved the final manuscript.

## Pre-publication history

The pre-publication history for this paper can be accessed here:

http://www.biomedcentral.com/1471-2431/14/43/prepub
